# Identification of Nine Novel Loci Associated with White Blood Cell Subtypes in a Japanese Population

**DOI:** 10.1371/journal.pgen.1002067

**Published:** 2011-06-30

**Authors:** Yukinori Okada, Tomomitsu Hirota, Yoichiro Kamatani, Atsushi Takahashi, Hiroko Ohmiya, Natsuhiko Kumasaka, Koichiro Higasa, Yumi Yamaguchi-Kabata, Naoya Hosono, Michael A. Nalls, Ming Huei Chen, Frank J. A. van Rooij, Albert V. Smith, Toshiko Tanaka, David J. Couper, Neil A. Zakai, Luigi Ferrucci, Dan L. Longo, Dena G. Hernandez, Jacqueline C. M. Witteman, Tamara B. Harris, Christopher J. O'Donnell, Santhi K. Ganesh, Koichi Matsuda, Tatsuhiko Tsunoda, Toshihiro Tanaka, Michiaki Kubo, Yusuke Nakamura, Mayumi Tamari, Kazuhiko Yamamoto, Naoyuki Kamatani

**Affiliations:** 1 Laboratory for Statistical Analysis, Center for Genomic Medicine (CGM), Institute of Physical and Chemical Research (RIKEN), Yokohama, Japan; 2 Department of Allergy and Rheumatology, Graduate School of Medicine, University of Tokyo, Tokyo, Japan; 3 Laboratory for Respiratory Diseases, Center for Genomic Medicine, Institute of Physical and Chemical Research, Yokohama, Japan; 4 Centre d'Etude du Polymorphisme Humain (CEPH), Paris, France; 5 Laboratory for Genotyping Development, Center for Genomic Medicine, Institute of Physical and Chemical Research, Yokohama, Japan; 6 Laboratory of Neurogenetics, National Institute on Aging, National Institutes of Health, Baltimore, Maryland, United States of America; 7 National Heart, Lung, and Blood Institute's (NHLBI) Framingham Heart Study, Framingham, Massachusetts, United States of America; 8 Department of Neurology, Boston University School of Medicine, Boston, Massachusetts, United States of America; 9 Department of Epidemiology, Erasmus MC, Rotterdam, The Netherlands; 10 Netherlands Consortium for Healthy Aging (NGINCHA), The Netherlands Genomics Initiative, Leiden, The Netherlands; 11 Icelandic Heart Association, Kopavogur, Iceland; 12 Longitudinal Studies Section, Clinical Research Branch, National Institute on Aging, National Institutes of Health, Baltimore, Maryland, United States of America; 13 Collaborative Studies Coordinating Center, Department of Biostatistics, The University of North Carolina at Chapel Hill, Chapel Hill, North Carolina, United States of America; 14 Department of Medicine, University of Vermont College of Medicine, Burlington, Vermont, United States of America; 15 Clinical Research Branch, National Institute on Aging, National Institutes of Health, Baltimore, Maryland, United States of America; 16 Laboratory for Epidemiology, Demography, and Biometry, National Institute on Aging, National Institutes of Health, Baltimore, Maryland, United States of America; 17 Division of Intramural Research, National Heart, Lung, and Blood Institute, Bethesda, Maryland, United States of America; 18 Division of Cardiovascular Medicine, Department of Internal Medicine, University of Michigan, Ann Arbor, Michigan, United States of America; 19 Laboratory of Molecular Medicine, Human Genome Center, Institute of Medical Science, University of Tokyo, Tokyo, Japan; 20 Laboratory for Medical Informatics, Center for Genomic Medicine, Institute of Physical and Chemical Research, Yokohama, Japan; 21 Laboratory for Cardiovascular Diseases, Center for Genomic Medicine, Institute of Physical and Chemical Research, Yokohama, Japan; University of Alabama at Birmingham, United States of America

## Abstract

White blood cells (WBCs) mediate immune systems and consist of various subtypes with distinct roles. Elucidation of the mechanism that regulates the counts of the WBC subtypes would provide useful insights into both the etiology of the immune system and disease pathogenesis. In this study, we report results of genome-wide association studies (GWAS) and a replication study for the counts of the 5 main WBC subtypes (neutrophils, lymphocytes, monocytes, basophils, and eosinophils) using 14,792 Japanese subjects enrolled in the BioBank Japan Project. We identified 12 significantly associated loci that satisfied the genome-wide significance threshold of *P*<5.0×10^−8^, of which 9 loci were novel (the *CDK6* locus for the neutrophil count; the *ITGA4*, *MLZE*, *STXBP6* loci, and the MHC region for the monocyte count; the *SLC45A3-NUCKS1*, *GATA2*, *NAALAD2*, *ERG* loci for the basophil count). We further evaluated associations in the identified loci using 15,600 subjects from Caucasian populations. These WBC subtype-related loci demonstrated a variety of patterns of pleiotropic associations within the WBC subtypes, or with total WBC count, platelet count, or red blood cell-related traits (n = 30,454), which suggests unique and common functional roles of these loci in the processes of hematopoiesis. This study should contribute to the understanding of the genetic backgrounds of the WBC subtypes and hematological traits.

## Author Summary

White blood cells (WBCs) are blood cells that mediate immune systems and defend the body against foreign microorganisms. It is well known that WBCs consist of various subtypes of cells with distinct roles, although the genetic background of each of the WBC subtypes has yet to be examined. In this study, we report genome-wide association studies (GWAS) for the 5 main WBC subtypes (neutrophils, lymphocytes, monocytes, basophils, and eosinophils) using 14,792 Japanese subjects. We identified 12 significantly associated genetic loci, and 9 of them were novel. Evaluation of the associations of these identified loci in cohorts of Caucasian populations demonstrated both ethnically common and divergent genetic backgrounds of the WBC subtypes. These loci also indicated a variety of patterns of pleiotropic associations within the hematological traits, including the other WBC subtypes, total WBC count, platelet count, or red blood cell-related traits, which suggests unique and common functional roles of these loci in the processes of hematopoiesis.

## Introduction

White blood cells (WBCs) mediate immune systems, and play essential roles in defending the body against invading foreign microorganisms [1]. WBCs consist of a variety of cells that mediate diverse roles, and are morphologically classified into 5 main subtypes: neutrophils, lymphocytes, monocytes, basophils, and eosinophils [1]. A number of previous studies have demonstrated significant contributions of these WBC subtypes to the regulation of innate and adaptive immune systems [2]–[6]. Since the number of WBC subtypes circulating in peripheral blood are tightly regulated, and abnormality in their numbers are closely linked to the presence and prognosis of diseases [2]–[6], the counts of WBC subtypes are widely used as important blood markers in medical treatment. Therefore, elucidation of the mechanism(s) that regulates the counts of WBC subtypes would have substantial clinical impact and would provide new insights into the etiology of the immune system.

WBC subtypes are known to be heritable traits and several epidemiological studies have suggested the existence of genetic factors that explain the variations in the counts of WBC subtypes, as well as a number of common environmental factors such as age, sex, and smoking [7]–[10]. Recently, genome-wide association studies (GWAS) have identified a number of genetic loci that affect hematological traits, but most of these identified loci were determined to be associated with red blood cell (RBC) or platelet (PLT) -related traits or total WBC count [11]–[17]. However, studies investigating WBC subtypes have yet to be further assessed [18]–[21]. Moreover, it is also of interest to evaluate whether ethnic differences underlie the genetic backgrounds that affect hematological traits.

Previous studies for hematological traits have also suggested that several genetic loci have pleiotropic associations with other hematological traits [15]–[18]. Therefore, it is of interest whether the WBC subtype-associated genetic loci have pleiotropic associations with counts of other WBC subtypes, RBCs, and PLTs, when considering the biological roles of the loci in the processes of hematopoiesis.

In this study, we report a large-scale GWAS for the counts of the WBC subtypes in 14,792 Japanese subjects enrolled in the BioBank Japan Project [22]. Subsequently, we performed pleiotropic association analysis of the identified WBC subtype-associated loci. We evaluated the associations of the loci identified in the Japanese population using data obtained by cohorts of Caucasian populations [23], in order to highlight the ethnically common and divergent genetic backgrounds of WBC subtypes.

## Results

### GWAS

In the GWAS for the WBC subtypes, we enrolled 8,794 Japanese subjects. The counts of the 5 main WBC subtypes (neutrophils, lymphocytes, monocytes, basophils, and eosinophils) of the subjects were collected from medical records and summarized in Table S1. We found moderate degree of correlation (*R^2^*>0.1) between the neutrophil and monocyte counts, between the basophil and lymphocyte counts, and between the basophil and eosinophil counts (Table S2). These results were considered to be compatible with previous reports [7]–[9]. To relatively compare the effect sizes on the traits, we carried out normalization of the counts of the respective WBC subtypes. The subjects with normalized values beyond ±4SD were discarded, which accounted for less than 0.5% of the total subjects.

Genotyping was performed with over 590,000 SNP markers using Illumina610-Quad Genotyping BeadChip (Illumina, CA, USA). We applied stringent quality control criteria, including principal component analysis (PCA) [24] to evaluate potential population stratification, and obtained genotype data for 481,110 autosomal SNPs. To extend the genomic coverage, we subsequently performed the whole-genome imputation of the SNPs, using HapMap Phase II genotype data of Japanese (JPT) and Han Chinese (CHB) individuals as references [25]. After the imputation, 2,178,645 autosomal SNPs that satisfied the criteria of a minor allele frequency (MAF) ≥0.01 and an imputation score (*Rsq* value by MACH software [26]) ≥0.7 were obtained. The associations of these imputed SNPs with the transformed values of the counts of WBC subtypes were evaluated using a linear regression model.

**Figure 1:**
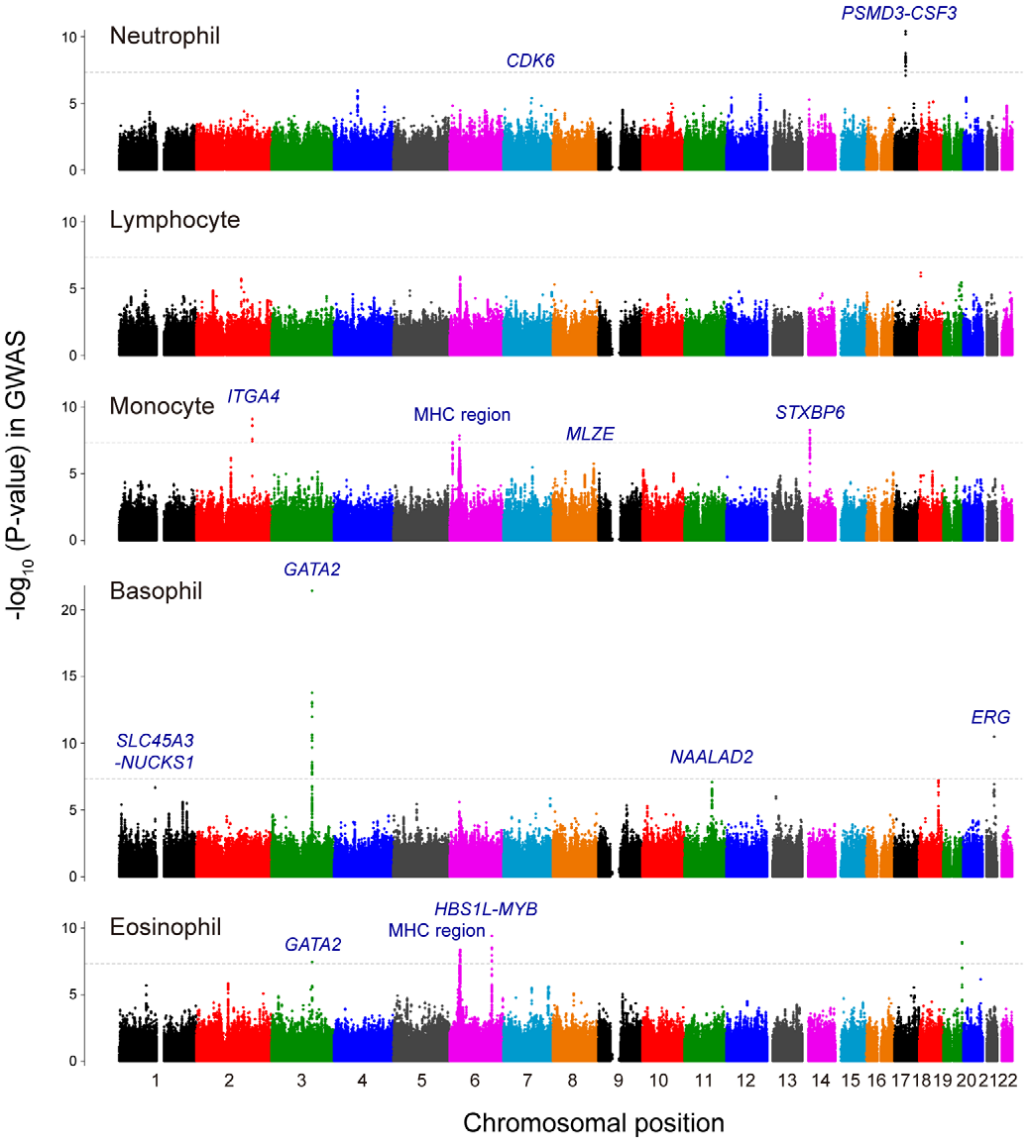
Manhattan plots of the GWAS for the WBC subtypes. Manhattan plots showing the -log_10_ (P-values) of the SNPs in the GWAS for neutrophil, lymphocyte, monocyte, basophil, and eosinophil counts. The genetic loci that satisfied the genome-wide significance threshold of *P*<5.0×10^−8^ in the combined study of the GWAS and the replication study were labeled in each of the traits. The gray horizontal line represents the threshold of *P* = 5.0×10^−8^.

Quantile-Quantile plots of P-values indicated remarkable departures from the null hypothesis in their tails, except for the lymphocyte count (Figure S1). Inflation factors of P-values, λ_GC_ [27], were as low as 1.024–1.038, which suggested no substantial population stratification existed in our study population as previously anticipated for the Japanese population [28]. We identified 10 significant associations that satisfied the genome-wide significance threshold of *P*<5.0×10^−8^ in the GWAS for the counts of neutrophils, monocytes, basophils and eosinophils (Figure 1 and Table S3). We also evaluated the associations in the previously-reported WBC subtype-associated loci, and observed significant associations in six of these loci (the *PSMD3-CSF3* and *PLCB4* loci for the neutrophil counts [21], the MHC region for the lymphocyte counts [20], the *IL1RL1*, *IKZF2*, *HBS1L-MYB* loci for the eosinophil counts [18], *P*<0.005; Table S4).

### Combined analysis of GWAS and replication study

We subsequently performed a replication study using independent 5,998 Japanese subjects, and further evaluated the associations of the loci by combining the results of the GWAS and the replication study. We selected a total of 36 genetic loci that showed *P*<5.0×10^−6^ in the GWAS for any of the WBC subtypes as the candidates for inclusion in the replication study. As a result of combined study, we finally identified 12 genetic loci that satisfied genome-wide significance threshold of *P*<5.0×10^−8^. Of these, the top-associated SNPs in 2 loci were genotyped and the SNPs in 10 loci were imputed with imputation score of *Rsq*>0.90. Specifically, we found 2, 4, 4, and 3 loci for neutrophil, monocyte, basophil, and eosinophil counts, respectively (Table 1, Table S3, and Figure 1). One locus was shared between basophil and eosinophil counts. On the other hand, no significant association was detected for the lymphocyte count in the combined study.

**Table 1:** Significantly associated SNPs in the GWAS for WBC subtypes.

					EA/	Japanese population								Caucasian population			
			Cyto		non	GWAS (n = 8,794)				Validation (n = 5,998)		Combined (n = 14,792)		CHARGE (n = 15,600)			
rsID^a^	Chr	Position	-band	Gene	EA^b^	Freq.^c^	Rsq^d^	Beta (SE)^e^	P	Beta (SE)^e^	P	Beta (SE)^e^	P	Freq.^c^	Beta (SE)^f^	P	ref.
GWAS for neutrophil count																	
rs445	7	92,246,306	7q21	*CDK6*	C/T	0.69	-	0.074 (0.016)	4.3×10^−6^	0.078 (0.019)	4.5×10^−5^	0.076 (0.012)	6.6×10^−10^	0.90	0.022 (0.009)	0.012	-
rs4794822	17	35,410,238	17q21	*PSMD3-CSF3*	T/C	0.52	1.00	0.099 (0.015)	4.5×10^−11^	0.085 (0.018)	1.6×10^−6^	0.093 (0.011)	4.0×10^−16^	0.46	0.043 (0.004)	3.6×10^−23^	[21]
GWAS for monocyte count																	
rs12988934	2	182,031,910	2q31	*ITGA4*	T/C	0.27	0.93	0.116 (0.019)	8.4×10^−10^	0.100 (0.021)	3.5×10^−6^	0.109 (0.014)	2.0×10^−14^	0.07	0.034 (0.010)	0.0010	-
rs3095254	6	31,329,647	6p21	MHC region	C/G	0.46	0.90	0.085 (0.015)	1.5×10^−8^	0.060 (0.017)	5.1×10^−4^	0.074 (0.011)	5.6×10^−11^	0.38	0.008 (0.005)	0.12	-
rs10956483	8	130,641,292	8q24	*MLZE*	C/G	0.44	0.97	0.070 (0.015)	2.0×10^−6^	0.072 (0.017)	1.6×10^−5^	0.071 (0.011)	2.1×10^−10^	0.15	0.012 (0.006)	0.072	-
rs10147992	14	24,573,639	14q12	*STXBP6*	G/A	0.47	0.98	0.084 (0.014)	5.5×10^−9^	0.029 (0.017)	0.085	0.061 (0.011)	1.2×10^−8^	0.35	0.008 (0.005)	0.099	-
GWAS for basophil count																	
rs12748961	1	203,942,886	1q32	*SLC45A3-NUKS1*	T/C	0.51	0.95	0.073 (0.015)	2.6×10^−6^	0.050 (0.019)	0.0069	0.064 (0.012)	4.2×10^−8^	0.97	0.002 (0.002)	0.33	-
rs4328821	3	129,799,125	3q21	*GATA2*	A/G	0.66	1.00	0.154 (0.016)	4.4×10^−22^	0.171 (0.019)	1.1×10^−19^	0.161 (0.012)	5.3×10^−40^	0.90	0.010 (0.002)	2.6×10^−8^	-
rs11018874	11	89,515,085	11q14	*NAALAD2*	G/A	0.69	0.96	0.089 (0.017)	8.2×10^−8^	0.062 (0.020)	0.0017	0.077 (0.013)	1.8×10^−9^	0.87	0.001 (0.002)	0.69	-
rs7275212	21	38,774,421	21q22	*ERG*	T/A	0.08	0.93	0.191 (0.029)	3.5×10^−11^	0.205 (0.035)	6.4×10^−9^	0.197 (0.022)	1.6×10^−18^	0.02	0.016 (0.008)	0.063	-
GWAS for eosinophil count																	
rs4328821	3	129,799,125	3q21	*GATA2*	A/G	0.66	1.00	0.087 (0.016)	3.9×10^−8^	0.125 (0.019)	3.6×10^−11^	0.103 (0.012)	3.3×10^−17^	0.90	0.014 (0.003)	6.7×10^−7^	[18]
rs2516399	6	31,589,278	6p21	MHC region	A/G	0.81	-	0.111 (0.019)	4.4×10^−9^	0.095 (0.024)	7.0×10^−5^	0.105 (0.015)	1.8×10^−12^	0.94	0.004 (0.004)	0.36	[18]
rs9373124	6	135,464,902	6q23	*HBS1L-MYB*	T/C	0.65	0.96	0.101 (0.016)	4.1×10^−10^	0.048 (0.020)	0.015	0.080 (0.012)	1.3×10^−10^	0.76	0.005 (0.002)	0.013	[18]

**^a^**SNPs that satisfied genome-wide significance threshold of *P*<5.0×10^−8^ in the combined study in Japanese population were indicated.

**^b^**The allele that increased the count of the corresponding WBC subtype was denoted as effect allele (EA) and is indicated based on the forward strand.

**^c^**Frequency of allele 1.

**^d^**Imputation score of *Rsq* by MACH 1.0 [26]. For the genotyped SNP, “-” is indicated.

**^e^**Effect size of effect allele on the normalized trait.

**^f^**Effect size of effect allele on the transformed trait. Details of the study by the CHARGE Consortium is summarized in Table S5 and described elsewhere [23].

GWAS, Genome-wide association study; WBC, white blood cell; EA, effect allele; SE, standard error.

**Figure 2:**
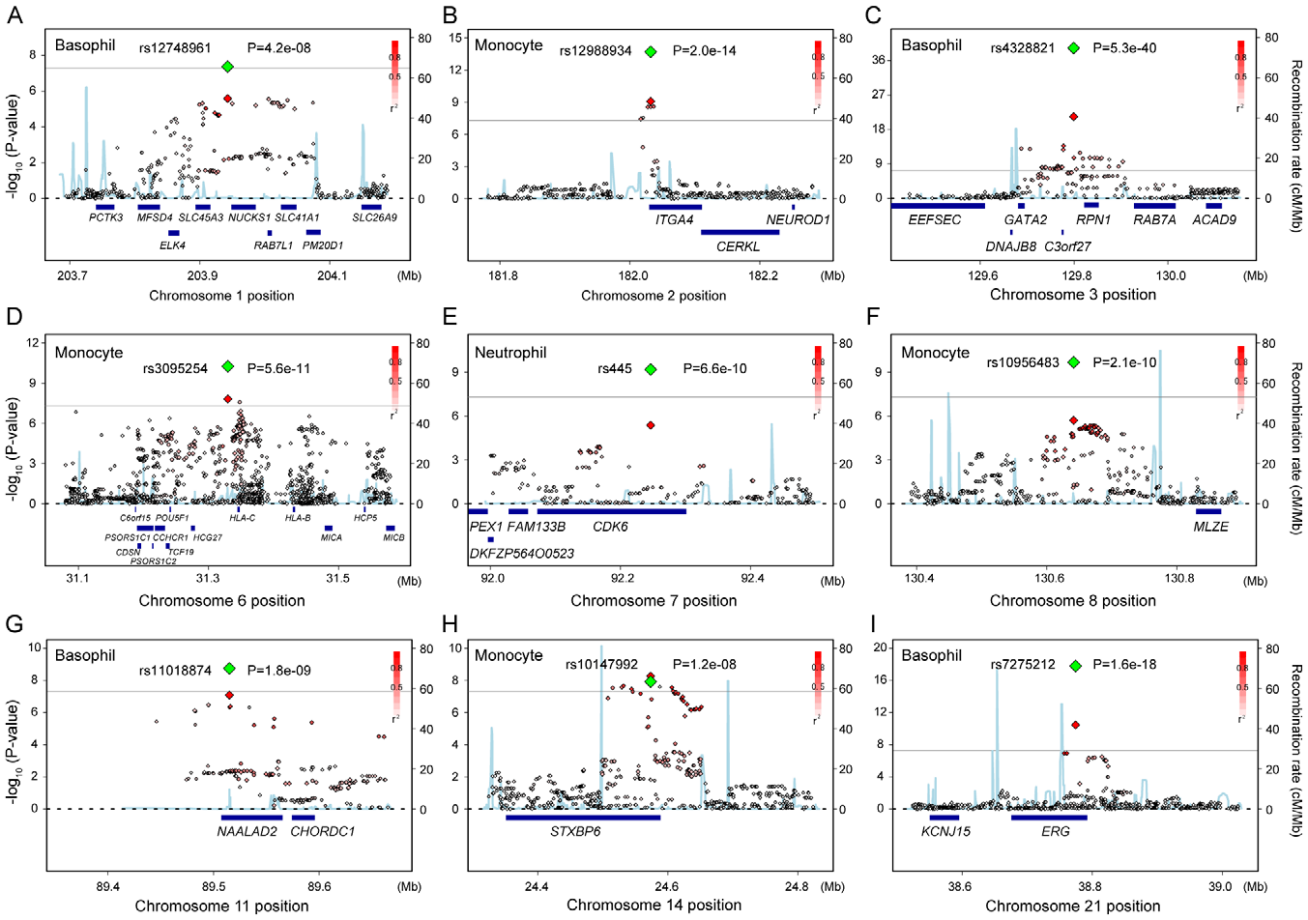
Regional plots of the novel genetic loci associated with the WBC subtypes. (A–I) Regional plots of the SNPs in the 9 novel loci identified in the GWAS for neutrophil, lymphocyte, monocyte, basophil, and eosinophil counts. Diamond-shaped dots represent -log_10_ (P-values) of the SNPs. The green dot indicates the P-value of the most significantly associated SNP in each of the loci in the combined study, and the red dot indicates its P-value in the GWAS. The density of the red color in the small-sized dots represents the *r^2^* value with the most significantly associated SNP of the large-sized red dot. The blue line shows the recombination rates given by the HapMap database. The gray horizontal line represents the genome-wide significance threshold of *P* = 5.0×10^−8^. The lower part indicates the RefSeq genes in the locus. The plots were drawn using SNAP, version 2.1 [48].

Among the identified loci in the combined study, 4 loci were the replication for the previous studies: rs4794822 in the *PSMD3-CSF3* locus for the neutrophil count [21], rs4328821 in the *GATA2* locus, rs2516399 in the MHC region, and rs9373124 in the *HBS1L-MYB* locus for the eosinophil count [18]. Associations in the other 9 loci were novel findings to our knowledge (Figure 2). Specifically, we identified associations in one locus for the neutrophil count (rs445 in the *CDK6* locus), 4 loci for the monocyte counts (rs12988934 in the *ITGA4* locus, rs3095254 in the MHC region, rs10956483 in the *MLZE* locus, and rs10147992 in the *STXBP6* loci), and 4 loci for the basophil count (rs12748961 in the *SLC45A3-NUCKS1* locus, rs4328821 in the *GATA2* locus, rs11018874 in the *NAALAD2* locus, and rs7275212 in the *ERG* loci) (Figure 2). Of the associated SNPs located in the MHC region, rs3095254 was reported to be in linkage disequilibrium (LD) with HLA-Cw*0702 allele (*D*′ = 1 and *r^2^* = 0.24), and rs2516399 was in LD with HLA-DRB1*0405 and HLA-DQB1*0401(*D*′>0.7 and *r^2^*>0.2) [29].

### Associations of the identified loci in Caucasian populations

To highlight the ethnically common and divergent genetic backgrounds of the WBC subtypes, the associations of the 12 identified loci were further evaluated in Caucasian populations by using 15,600 subjects in cohorts of the CHARGE Consortium (Table S5) [23]. The CHARGE Consortium consists of multiple community-based and prospectively designed cohorts from the United States and Europe [30] and has performed association studies for hematological traits, including the WBC subtypes [23]. We observed the same directional effects of the alleles in all 12 loci evaluated in the CHARGE Consortium. Furthermore, significant associations were observed in 4 loci (the *PSMD3-CSF3* locus for the neutrophil count, the *ITGA4* locus for the monocyte count, and the *GATA2* locus for both the basophil and eosinophil counts; *P*<0.004). We also observed the suggestive associations in 2 loci (the *CDK6* locus for the neutrophil count and the *HBS1L-MYB* locus for the eosinophil count; *P*<0.05).

### Pleiotropic association study for WBC subtype-associated loci

We next evaluated the pleiotropic associations of the WBC subtype-associated loci. For the top-associated SNPs in each of the loci that indicated significant associations in our study (*P*<5.0×10^−8^), we evaluated the associations with the counts of the other WBC subtypes, total WBC count, RBC-related traits (RBC count, hemoglobin [Hb], hematocrit [Ht], mean corpuscular hemoglobin [MCH], mean corpuscular hemoglobin concentration [MCHC], mean corpuscular volume [MCV]), and PLT count using 30,454 Japanese subjects (Table S6).

**Figure 3:**
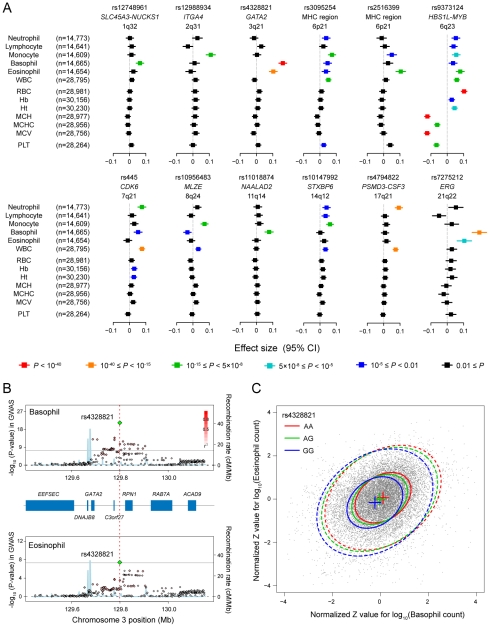
Pleiotropic associations of the genetic loci associated with the WBC subtypes. (A) Associations of the SNPs identified in the GWAS for the WBC subtypes with other WBC subtypes and hematological traits. Effect sizes of each SNP on the normalized traits are aligned vertically in each of the loci, and their color corresponds to the P-values of the associations according to the legend. WBC, total white blood cell count; RBC, red blood cell count; Hb, hemoglobin; Ht, hematocrit; MCV, mean corpuscular volume; MCH, mean corpuscular hemoglobin; MCHC, mean corpuscular hemoglobin concentration; PLT, platelet count; CI confidence interval. (B) Regional plots of the SNPs in the GWAS for the basophil (upper) and eosinophil (lower) counts in the *GATA2* locus. Diamond-shaped dots represent -log_10_ (P-values) of the SNPs in the GWAS; green indicates the most significantly associated SNP, and the density of the red color represents the *r^2^* value with the most significantly associated SNP. The blue line shows recombination rates given by the HapMap database. The gray horizontal line represents the genome-wide significance threshold of *P* = 5.0×10^−8^. The middle part indicates the RefSeq genes in the locus. The red vertical dashed line represents the concordance of the peaks of the association at rs4328821 in the GWAS. (C) Scatter plot of the subjects enrolled in the GWAS and the replication study based on the normalized Z values for the basophil and eosinophil counts. The center, 50% probable ellipse, and 95% probable ellipse of the subjects with AA (red) / AG (green) / GG (blue) genotypes of rs4328821 are indicated as crosses, solid ellipses, and dashed ellipses, respectively.

**Figure 4:**
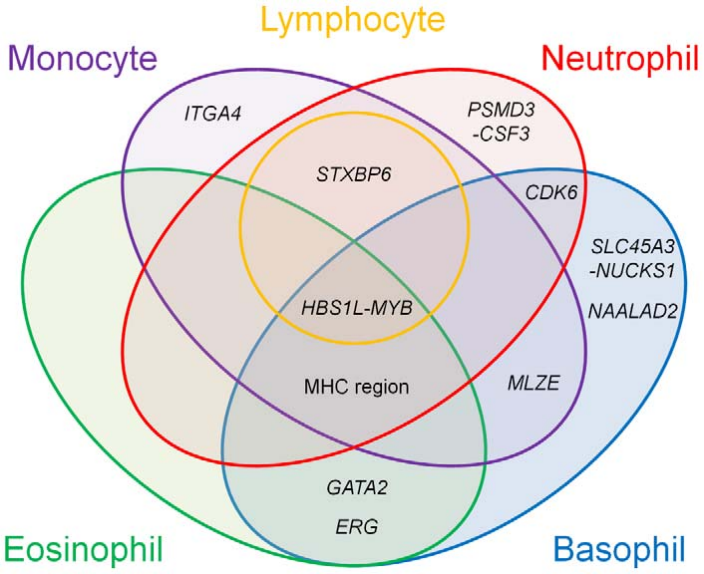
Venn diagram of the pleiotropic associations among the WBC subtypes. Genetic loci identified in the GWAS for the WBC subtypes are classified based on the results of the pleiotropic association study among the WBC subtypes. The colors in the Venn diagram (red, orange, purple, aqua, and green) correspond to each of the WBC subtypes (neutrophils, lymphocytes, monocytes, basophils, and eosinophils, respectively). Pleiotropic associations with *P*<0.01 are included.

We found various patterns of pleiotropic associations for the loci associated with the WBC subtypes (Figure 3A and Figure 4). Three loci demonstrated specific associations with the original WBC subtypes identified in the GWAS (rs12748961 in the *SLC45A3-NUCKS1* locus, rs12988934 in the *ITGA4* locus, rs11018874 in the *NAALAD2* locus), although 9 other loci demonstrated pleiotropic associations with other traits. The most pleiotropic associations were observed in the *HBS1L-MYB* locus, which indicated significant associations with all of the evaluated hematological traits (*P*<0.005). The T allele of rs9373124 that increased the eosinophil count also increased the counts of the other WBC subtypes, total WBC count, RBC count, the Hb and Ht levels, and conversely decreased MCH, MCHC, MCV, and PLT count, validating its substantial role in hematopoiesis [15], [31].

In the *GATA2* locus, we observed significant associations with both the basophil and eosinophil counts (*P*<5.0×10^−8^; Figure 3B). This locus encompassed several genes, although *GATA2* seemed to be most responsible for regulating eosinophils and basophils from a functional standpoint [3], [32]. Interestingly, the peaks of the associations of the SNPs in the basophil and eosinophil GWAS showed concordance at rs4328821. Possession of the A allele of rs4328821 increased both the basophil and eosinophil counts (Figure 3C). The subjects who were homozygous for the A allele had 1.28-fold and 1.19-fold higher basophil and eosinophil counts, respectively, compared with the corresponding levels of the subjects who were homozygous for the G allele. Moreover, rs4328821 significantly explained 2.7% of the correlation between the basophil and eosinophil counts (permutation *P*<1.0×10^−9^). Upon combining the effects of the SNPs in the identified loci, up to 2.1% of the variations of the counts of the WBC subtypes was explained, and up to 8.0% of the correlation between the WBC subtypes was explained (Table S2).

## Discussion

Through a GWAS and a replication study consisting of 14,792 Japanese subjects, our study identified 12 loci that were significantly associated with the counts of WBC subtypes. Among the identified loci, 9 loci are reported for the first time in this study. The identified loci demonstrated a variety of patterns of pleiotropic associations within the WBC subtypes and/or with total WBC count, RBC-related traits, and PLT count, which suggest they have both unique and common roles in the processes of hematopoiesis. Comparison of the loci identified in the Japanese population with those in Caucasian populations demonstrated the ethnically common and divergent genetic backgrounds of the various WBC subtypes.

Two loci identified in the GWAS for the neutrophil count (the *CDK6* and *PSMD3-CSF3* loci) have previously been reported to be associated with the total WBC count [16], [17]. Since neutrophils are the most abundant subtype of WBCs and typically comprise 50–70% of the total WBC count, the associations between these loci with the total WBC count would have reflected the associations with the neutrophil count [21].

We identified 4 novel loci associated with monocyte count (the *ITGA4*, *MLZE*, and *STXBP6* locus and the MHC region). The landmark SNP in the MHC region was located near the *HLA-C* gene and in moderate LD with the particular HLA-C allele, which belongs to MHC class I molecules. *ITGA4* encodes the α4 chain of the integrins, which mediate migration of the WBCs [33]. Previous reports have demonstrated that *STXBP6* (also known as *amisyn*) binds to the components of the SNARE complex, which mediates membrane fusions including phagocytosis [34], [35]. In response to inflammation, monocytes differentiate into macrophages and migrate into affected tissues of inflammation, and subsequently perform phagocytosis and antigen presentation using MHC molecules expressed on the cell surface [6]. Therefore, associations of the SNPs in these loci with the monocyte count would be plausible from a biological perspective. Recently, clinical benefits of inhibition of α4 integrin have been demonstrated in the treatment of autoimmune diseases [36]. Although further functional investigation is necessary, the SNP in the *ITGA4* locus that was identified in our GWAS may be a promising target for pharmacogenomics of anti-α4 integrin therapy. *MLZE* (also known as *GCDMC*) belongs to the Gasdermin family of genes [37], and its role in the regulation of the monocyte count should be further explored.

In addition, we identified 4 novel loci associated with the basophil count (the *SLC45A3-NUCKS1*, *GATA2*, *NAALAD2*, and *ERG* loci) and replicated associations in 3 previously-reported loci associated with the eosinophil count (the *GATA2* and *HBS1L-MYB* loci and the MHC region). Basophils and eosinophils coordinately mediate allergic inflammation [3]–[5], and the correlation of these counts [7] suggested the existence of genetic factors that are shared between them. Our pleiotropic study demonstrated overlap of the associated loci between the basophil and eosinophil counts, which was most highlighted in the *GATA2* locus. *GATA2* is a well-known zinc-finger transcription factor and plays an essential role in hematopoiesis, particularly in the regulation of basophils and eosinophils [3], [32]. The landmark SNP in the *GATA2* locus was concordant in the GWAS for both the basophil and eosinophil counts, and significantly explained part of their correlation in the counts. Pleiotropic associations of the SNP with the basophil and eosinophil counts were further replicated in the Caucasian populations. These results suggested an ethnically-shared substantial functional role of the SNP in the etiology of *GATA2*.

*ERG* encodes a member of the Ets family of transcription factors, and is known to be included in the Down syndrome critical region on chromosome 21 [38]. Although its functional role(s) in the regulation of basophils has not been investigated to date, an essential role of *ERG* for definitive hematopoiesis has been demonstrated [38]. The *SLC45A3-NUCKS1* locus in the present study encompassed several genes, and we submit that the functional origin of this locus should be further investigated. Interestingly, the fusion transcript of *SLC45A3* and *ERG* is observed in prostate cancers, which have been characterized by the overexpression of *ERG* mRNA [39], [40], although we did not find any significant gene-gene interaction in the SNPs in these two loci for basophil count (data not shown). Finally, *NAALAD2* is a member of the *N*-acetylated α-linked acidic dipeptidase gene family [41], and its role in the regulation of the basophil counts should be a topic to be further investigated in the future.

In contrast to the WBC subtypes mentioned above, no significant association was detected for the lymphocyte count. One probable explanation for this finding is that lymphocytes can be further divided into a variety of subsets, such as natural killer (NK) cells, T cells, and B cells, which were not examined specifically in this study. Therefore, future GWAS that focus on those specific subsets of lymphocytes [20] are necessary to efficiently investigate the genetic backgrounds of the lymphocytes.

Several points about this study bear discussion. First, since our study populations consisted of the disease patients, it would be useful to assess the possibility that the disease status might have confounded the results. When the respective disease groups were analyzed separately and evaluated through meta-analysis, all the identified loci satisfied the significant associations (*P*<5.0×10^−8^) without significant heterogeneities of the effects (α = 0.01). None of the identified loci has been reported to be associated with the risk of the diseases enrolled in the study population, except for the MHC region with Rheumatoid Arthritis (RA) [42]. Moreover, after the subjects affected with RA were excluded, the significant associations of the SNPs in the MHC region with the monocyte and eosinophil counts were observed (*P* = 1.7×10^−10^ for rs3095254 and *P* = 9.6×10^−12^ for rs2516399, respectively). It would be of note that we observed concordance of the associations in the identified loci with those by CHARGE consortium, which is consisted of multiple community-based and prospective cohorts incorporating normal populations. Although further validation study using non-affected subjects would be desirable, these observations suggested that the utilization of disease patients have not induced substantial bias in our study.

Second, the counts of the WBC subtypes were based on medical records. Although the data collection protocol is enormously standardized [22], there is a possibility that unstandardized discrepancy among the medical institutes might induce bias in the phenotype distributions and impair statistical power of the study.

Third, the explained proportion of the WBC subtypes by the identified loci would be estimated conservatively. Because of the stringent significance threshold adopted in the study, a number of associated loci with moderate effect sizes would be still unidentified. Further approaches, such as considering the entire SNPs simultaneously [43], are necessary for the accurate estimation of the explained variations. Candidate gene analysis based on the biological pathway of the WBC subtypes would also be a promising approach to uncover these unidentified loci [44].

Fourth, since the correlation among the traits could modulate the pattern of the pleiotropic associations, further distinction of actual pleiotropic associations from simple associations induced by the correlations would be a topic to be investigated.

In summary, our study identified 9 novel loci that are associated with the counts of the WBC subtypes. The pleiotropic association study of the identified loci demonstrated unique and common genetic backgrounds underlying the WBC subtypes. Our study should contribute to the general understanding of the etiology and regulation of the WBC subtypes.

## Materials and Methods

### Subjects

The subjects enrolled in the GWAS, and in the replication study for WBC subtypes (n = 14,792), and in the pleiotropic association study for hematological traits (n = 30,454) consisted of patients that were classified into 27 disease groups (Tables S1 and S6). The subjects in the pleiotropic association study included the subjects in the GWAS and the replication study. All subjects were collected under the support of the BioBank Japan Projects [22]. Subjects who were determined to be of non-Japanese origin by either self-report or by PCA in GWAS were excluded from analysis. Some of the subjects in this study have also been included in our previous studies [17], [21], [45], [46]. All participants provided written informed consent as approved by the ethical committees of the BioBank Japan Project [22] and the University of Tokyo. Clinical information of the subjects including age, gender, and smoking history were collected by self-report on the questionnaire. The laboratory data including the counts of the WBC subtypes and other hematological traits were collected from medical records by the professional medical coordinators according to the standardized protocol [22]. The details of the study enrolled by the CHARGE Consortium, including subject details and the study design, are described at length elsewhere [23], and are summarized in Table S5.

### Genotyping and quality control

In the GWAS for the WBC subtypes, 592,232 SNPs were genotyped for 8,943 subjects using Illumina HumanHap610-Quad Genotyping BeadChip. We excluded 77 subjects with call rates <0.98 in the process of genotyping. After this initial exclusion, SNPs with call rates <0.99 or with ambiguous clustering of the intensity plots, or non-autosomal SNPs, were excluded. We excluded 67 closely related subjects based on the identity-by-descent (IBD), which was estimated using the “–genome” option implemented in PLINK version 1.06 [47]. For each pair with a 1st or 2nd degree of kinship, we excluded the one member of the pair with lower call rates than the other. We then excluded subjects whose ancestries were estimated to be distinct from East-Asian populations using PCA performed by EIGENSTRAT version 2.0 [24]. We performed PCA for the genotype data of our GWAS along with the genotype data of Phase II HapMap populations (unrelated European (CEU), African (YRU), and East-Asian (JPT + CHB) individuals) (release 24) [25]. Based on the PCA plot of the subjects, we visually identified and excluded 5 outliers in terms of ancestry from JPT + CHB clusters. Subsequently, the SNPs with MAF <0.01 or with an exact P-value of the Hardy-Weinberg equilibrium test <1.0×10^−7^ were excluded. Finally, we obtained 481,110 SNPs for 8,794 subjects.

After the quality control criteria mentioned above were applied, genotype imputation was performed using MACH 1.0 [26] in a two-step procedure [46]. The genotype data of Phase II HapMap JPT and CHB individuals (release 24) [25] were adopted as references. In the first step of the imputation, recombination and error rate maps were estimated using 500 randomly selected subjects from those enrolled in the GWAS. In the second step, genotype imputation of all subjects was performed using the estimated recombination and error rate maps. Quality control filters of MAF ≥0.01 and *Rsq* values ≥0.7 were applied for the imputed SNPs.

The genotype data of the SNPs enrolled in the replication or pleiotropic association study were obtained from the genome-wide screening data of the BioBank Japan Project [22]. Genotyping was performed using either Illumina HumanHap550v3 Genotyping BeadChip or Illumina HumanHap610-Quad Genotyping BeadChip, and the same quality control filters and imputation procedure were applied.

### Statistical analysis

The common log-transformed values of the counts of each of the WBC subtypes were adjusted for gender, age, smoking history, and the affection statuses of the subjects with the disease groups (Table S1), using linear regression by *R* statistical software (version 2.11.0). Then the residuals were normalized as *Z* scores, and the subjects with Z score >4.0 or <−4.0 were excluded in each of the traits. Associations of the SNPs with the counts of the WBC subtypes were assessed by linear regression assuming the additive effects of the allele dosages on the *Z* scores, using mach2qtl software [26]. In the replication and pleiotropic association studies, the association of the SNPs with the normalized residuals were also evaluated by the linear regression as univariate analysis for each of the phenotypes. The transformation methods used for the hematological traits in the pleiotropic association study are summarized in Table S6. Combined study of the results of the GWAS and the replication study was performed using an inverse-variance method from the summary statistics of beta and standard error (SE). Through the combined study of the GWAS and the replication study, the locus which satisfied the genome-wide significance threshold of *P*<5.0×10^−8^ was considered to be significant. We did not account for multiple comparisons among the traits. These significantly associated loci were subsequently enrolled in the pleiotropic association study. For the selection of the loci that were evaluated in the replication study, we adopted less stringent threshold of *P*<5.0×10^−6^ to include potentially associated loci. For the evaluation of the identified loci using the Caucasian populations, Bonferroni correction based on the number of the evaluated loci were adopted (α = 0.05, n = 12, *P*<0.004). LD between the SNPs in the MHC region and HLA alleles were estimated using the genotype data of the SNPs and the high-resolution HLA alleles for Phase II HapMap JPT and CHB individuals [25], [29]. Explained proportions of the variations of the WBC subtypes by the combination of the associated SNPs were estimated based on the differences of the coefficient of determination, *R^2^*, in the multivariate linear regression model for common-log transformed counts of the respective WBC subtypes, including the associated SNPs as covariates, and the model additionally including age, gender, smoking history, and the affection statuses of the subjects as covariates. Explained proportions of the correlation between the two WBC subtypes by the associated SNPs were estimated based on the following statistics: (*R^2^_resi1_* - *R^2^_resi2_*) / *R^2^_nomi_*, where *R^2^_nomi_* is the *R^2^* between the log-transformed values of the counts of the WBC subtypes, *R^2^_resi1_* is the *R^2^* between the residuals of the values adjusted for gender, age, smoking history, and the affection statuses of the subjects, and *R^2^_resi2_* is the *R^2^* between the residuals of the values adjusted for gender, age, smoking history, the affection statuses of the subjects, and the SNPs. Significance of the statistics was evaluated using permutation procedure (× 10^9^ iteration steps).

### Web resources

The URLs for data presented herein are as follows.

BioBank Japan Project, http://biobankjp.org

MACH and mach2qtl software, http://www.sph.umich.edu/csg/abecasis/MACH/index.html

International HapMap Project, http://www.hapmap.org

PLINK software, http://pngu.mgh.harvard.edu/~purcell/plink/index.shtml

EIGENSTRAT software, http://genepath.med.harvard.edu/~reich/Software.htm

*R* statistical software, http://cran.r-project.org

SNAP, http://www.broadinstitute.org/mpg/snap/index.php

## Supporting Information

Figure S1 Quantile-Quantile plots (QQ-plots) of P-values in the GWAS for the WBC subtypes. QQ-plots of the GWAS for (A) neutrophil, (B) lymphocyte, (C) monocyte, (D) basophil, and (E) eosinophil counts. The horizontal axis indicates the expected -log_10_ (P-values). The vertical axis indicates the observed -log_10_ (P-values). The gray line represents y  =  x. λ_GC_ represents the inflation factor of the test statistics. The SNPs for which the P-value was smaller than 1.0×10^−15^ are indicated at the upper limit of the plot.
(TIF)

Table S1 Characteristics and distributions of traits in the Japanese subjects.
(DOC)

Table S2 Correlation among the WBC subtypes and the proportions explained by the SNPs identified in the study.
(DOC)

Table S3 Results of genome-wide association studies for the WBC subtypes.
(DOC)

Table S4 The associations in the previously-reported WBC subtype-associated loci.
(DOC)

Table S5 Characteristics and distributions of traits in the study populations by the CHARGE Consortium.
(DOC)

Table S6 Characteristics and distributions of the traits enrolled in the pleiotropic association study.
(DOC)

Text S1 Full descriptions of acknowledgements.
(DOC)
