# Supplementary figures and images for: Identification of Nine Novel Loci Associated with White Blood Cell Subtypes in a Japanese Population
Source: PLoS Genet. 2011 Jun 30;7(6):e1002067. doi: 10.1371/journal.pgen.1002067 (PMC3128095; doi:10.1371/journal.pgen.1002067)

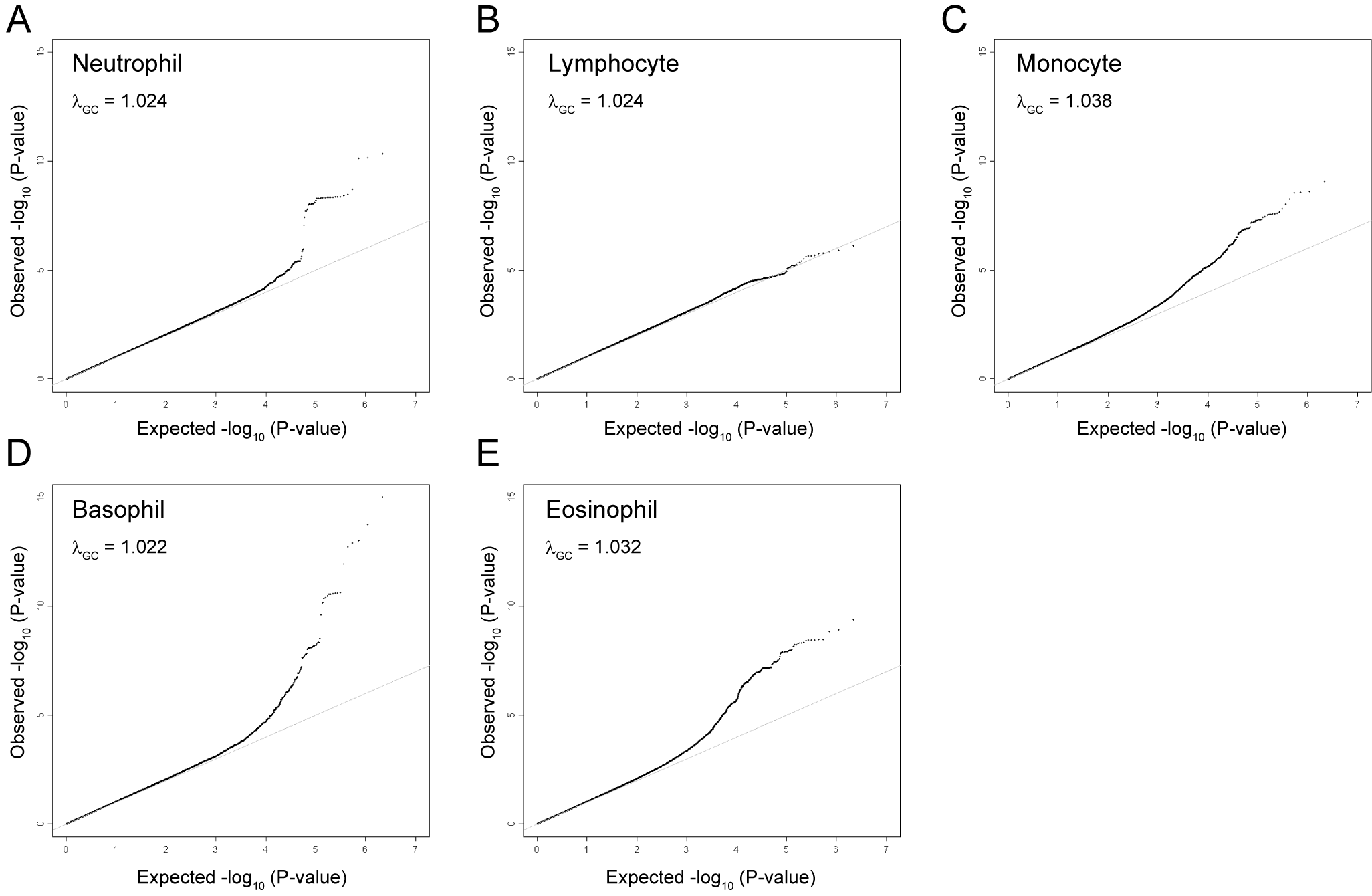

Supplement: Figure S1 — Quantile-Quantile plots (QQ-plots) of P-values in the GWAS for the WBC subtypes. QQ-plots of the GWAS for (A) neutrophil, (B) lymphocyte, (C) monocyte, (D) basophil, and (E) eosinophil counts. The horizontal axis indicates the expected -log10 (P-values). The vertical axis indicates the observed -log10 (P-values). The gray line represents y = x. λGC represents the inflation factor of the test statistics. The SNPs for which the P-value was smaller than 1.0×10^−15^ are indicated at the upper limit of the plot. (TIF) [file pgen.1002067.s001.tif]
