# Supplementary material for: Identification of Nine Novel Loci Associated with White Blood Cell Subtypes in a Japanese Population
Source: PLoS Genet. 2011 Jun 30;7(6):e1002067. doi: 10.1371/journal.pgen.1002067 (PMC3128095; doi:10.1371/journal.pgen.1002067)
Supplement: Table S1 — Characteristics and distributions of traits in the Japanese subjects. (DOC) [file pgen.1002067.s002.doc]

**Table S1.** Characteristics and distributions of traits in the Japanese subjects.

|  |  | Age | Gender | Smoking | Neutrophil^b^ | Lymphocyte^b^ | Monocyte^b^ | Basophil^b^ | Eosinophil^b^ |
| --- | --- | --- | --- | --- | --- | --- | --- | --- | --- |
| Stage | No. subjects^a^ | (mean ± SD) | (Female %) | (%) | (×10^3^/μL) | (×10^3^/μL) | (×10^3^/μL) | (×10^3^/μL) | (×10^3^/μL) |
| GWAS | 8,794 | 62.1 ± 11.0 | 46.3 | 42.2 | 3.94 ± 1.65 | 1.69 ± 0.61 | 0.38 ± 0.15 | 0.038 ± 0.029 | 0.163 ± 0.138 |
|  |  |  |  |  | (n=8,788) | (n=8,668) | (n=8,653) | (n=8,671) | (n=8,660) |
| Validation study | 5,998 | 59.0 ± 14.5 | 51.7 | 51.8 | 3.63 ± 1.74 | 1.69 ± 0.63 | 0.37 ± 0.17 | 0.035 ± 0.024 | 0.170 ± 0.145 |
|  |  |  |  |  | (n=5,985) | (n=5,973) | (n=5,956) | (n=5,994) | (n=5,994) |
| Combined-analysis | 14,792 | 60.8 ± 12.6 | 48.5 | 46.1 | 3.82 ± 1.70 | 1.69 ± 0.62 | 0.37 ± 0.16 | 0.037 ± 0.027 | 0.166 ± 0.141 |
|  |  |  |  |  | (n=14,773) | (n=14,641) | (n=14,609) | (n=14,665) | (n=14,654) |

^a^No. total subjects enrolled in the study. No. subjects enrolled in the study of respective white blood cell subtypes are indicated in parentheses.

^b^For each of the traits, the subjects with values beyond ± 4 SD after the normalization of the common-log transformed values were excluded.

GWAS, Genome-wide association study.

Subjects consisted of Japanese patients in 27 disease groups as follows: drug eruption (n = 1,943), rheumatoid arthritis (n = 1,820), lung cancer (n = 1,045), breast cancer (n = 1,004), ischemic stroke (n = 924), Diabetes Mellitus (n = 911), gastric cancer (n = 808), colorectal cancer (n = 770), prostate cancer (n = 712), liver cancer (n = 658), myocardial infarction (n = 626), warfarin intake (n = 591), cirrhosis (n = 575), endometriosis (n = 417), fibroid of uteris (n = 381), atrial fibrillation (n = 328), peripheral artery disease (n = 293), osteoporosis (n = 210), tuberculosis (n = 155), pancreatic cancer (n = 134), hepatitis B (n = 116), gallbladder/bile duct cancers (n = 104), uterine body cancer (n = 71), esophageal cancer (n = 65), cervical cancer of uteris (n = 58), ovarian cancer (n = 46), and keloid (n = 27).
