# Supplementary material for: Identification of Nine Novel Loci Associated with White Blood Cell Subtypes in a Japanese Population
Source: PLoS Genet. 2011 Jun 30;7(6):e1002067. doi: 10.1371/journal.pgen.1002067 (PMC3128095; doi:10.1371/journal.pgen.1002067)
Supplement: Table S2 — Correlation among the WBC subtypes and the proportions explained by the SNPs identified in the study. (DOC) [file pgen.1002067.s003.doc]

**Table S2.** Correlation among the WBC subtypes and the proportions explained by the SNPs identified in the study.

|  | Neutrophil | Lymphocyte | Monocyte | Basophil | Eosinophil |
| --- | --- | --- | --- | --- | --- |
|  | (n=14,773) | (n=14,641) | (n=14,609) | (n=14,665) | (n=14,654) |
| Neutrophil | - | 0.0036 | 0.179 | 0.037 | 0.0019 |
|  | (0.93%) | (4.83%) | (1.06%) | (0.00%) | (8.03%) |
| Lymphocyte |  | - | 0.060 | 0.105 | 0.070 |
|  |  | (0.36%) | (0.00%) | (0.04%) | (2.60%) |
| Monocyte |  |  | - | 0.040 | 0.028 |
|  |  |  | (1.21%) | (0.81%) | (1.35%) |
| Basophil |  |  |  | - | 0.117 |
|  |  |  |  | (2.13%) | (4.50%) |
| Eosinophil |  |  |  |  | - |
|  |  |  |  |  | (1.41%) |

The coefficients of determination, *R^2^*, between each pair of the common-log transformed values of WBC subtypes are indicated.

The proportions of *R^2^* explained by the combination of the significantly-associated SNPs listed in Table 1 are indicated in parentheses.

In the downward diagonal cells of the table, the explained proportions of the variance of common-log transformed value of each WBC traits by the significantly-associated SNPs are indicated in parentheses.

WBC, white blood cell; GWAS, Genome-wide association study.
