# Supplementary material for: Identification of Nine Novel Loci Associated with White Blood Cell Subtypes in a Japanese Population
Source: PLoS Genet. 2011 Jun 30;7(6):e1002067. doi: 10.1371/journal.pgen.1002067 (PMC3128095; doi:10.1371/journal.pgen.1002067)
Supplement: Table S3 — Results of genome-wide association studies for the WBC subtypes. (DOC) [file pgen.1002067.s004.doc]

**Table S3**. Results of genome-wide association studies for the WBC subtypes.

| WBC |  |  |  |  |  | EA/ | GWAS (n=8,794) | | | |  | Validation (n=5,998) | |  | Combined (n=14,792) | |
| --- | --- | --- | --- | --- | --- | --- | --- | --- | --- | --- | --- | --- | --- | --- | --- | --- |
| subtype | rsID^a^ | Chr | Position | Cytoband | Gene | non-EA^b^ | Freq.^c^ | *Rsq*^d^ | Beta (SE)^e^ | *P* |  | Beta (SE)^e^ | *P* |  | Beta (SE)^e^ | *P* |
| Basophil | rs11587438 | 1 | 6,554,018 | 1p36 | *TAS1R1* | T/C | 0.83 | 0.87 | 0.098 (0.015) | 4.2×10^-6^ |  | 0.041 (0.025) | 0.10 |  | 0.075 (0.016) | 3.7×10^-6^ |
| Eosinophil | rs6576878 | 1 | 87,477,619 | 1p22 | *LMO4* | G/A | 0.43 | - | 0.072 (0.015) | 2.1×10^-6^ |  | 0.024 (0.018) | 0.20 |  | 0.053 (0.012) | 6.0×10^-6^ |
| Basophil | rs12748961 | 1 | 203,942,886 | 1q32 | *SLC45A3-NUKS1* | T/C | 0.51 | 0.95 | 0.073 (0.015) | 2.6×10^-6^ |  | 0.050 (0.019) | 0.0069 |  | 0.064 (0.012) | 4.2×10^-8^ |
| Basophil | rs796486 | 1 | 218,952,235 | 1q41 | *C1orf115* | C/A | 0.42 | - | 0.071 (0.015) | 3.4×10^-6^ |  | -0.001 (0.018) | 0.94 |  | 0.042 (0.012) | 3.0×10^-4^ |
| Eosinophil | rs17027258 | 2 | 102,457,972 | 2q12 | *SLC9A4* | G/A | 0.37 | 0.99 | 0.075 (0.016) | 1.4×10^-6^ |  | 0.027 (0.019) | 0.16 |  | -0.055 (0.012) | 7.2×10^-6^ |
| Monocyte | rs7578982 | 2 | 111,553,009 | 2q13 | *ACOXL* | T/C | 0.55 | - | 0.071 (0.014) | 7.1×10^-7^ |  | 0.030 (0.016) | 0.063 |  | 0.054 (0.011) | 4.1×10^-7^ |
| Lymphocyte | rs1228080 | 2 | 146,319,129 | 2q22 | no gene | C/T | 0.79 | 0.85 | 0.093 (0.020) | 2.1×10^-6^ |  | 0.030 (0.023) | 0.19 |  | 0.066 (0.015) | 1.3×10^-5^ |
| Monocyte | rs12988934 | 2 | 182,031,910 | 2q31 | *ITGA4* | T/C | 0.27 | 0.93 | 0.116 (0.019) | 8.4×10^-10^ |  | 0.100 (0.021) | 3.5×10^-6^ |  | 0.109 (0.014) | 2.0×10^-14^ |
| Basophil | rs4328821 | 3 | 129,799,125 | 3q21 | *GATA2* | A/G | 0.66 | 1.00 | 0.154 (0.016) | 4.4×10^-22^ |  | 0.171 (0.019) | 1.1×10^-19^ |  | 0.161 (0.012) | 5.3×10^-40^ |
| Eosinophil | rs4328821 | 3 | 129,799,125 | 3q21 | *GATA2* | A/G | 0.66 | 1.00 | 0.087 (0.016) | 3.9×10^-8^ |  | 0.125 (0.019) | 3.6×10^-11^ |  | 0.103 (0.012) | 3.3×10^-17^ |
| Neutrophil | rs546829 | 4 | 75,175,236 | 4q13 | *CXCL2* | A/T | 0.43 | 0.97 | 0.075 (0.015) | 1.1×10^-6^ |  | 0.037 (0.018) | 0.043 |  | 0.060 (0.012) | 2.5×10^-7^ |
| Basophil | rs10040209 | 5 | 74,389,291 | 5q13 | *GCNT4* | G/A | 0.80 | 0.86 | 0.095 (0.021) | 3.9×10^-6^ |  | 0.014 (0.024) | 0.58 |  | 0.060 (0.016) | 1.5×10^-4^ |
| Monocyte | rs9968917 | 6 | 7,212,001 | 6p24 | *RREB1-SSR1* | A/C | 0.31 | 0.98 | 0.085 (0.016) | 4.6×10^-8^ |  | -0.001 (0.018) | 0.96 |  | 0.046 (0.012) | 9.2×10^-5^ |
| Monocyte | rs3095254 | 6 | 31,329,647 | 6p21 | MHC region | C/G | 0.46 | 0.90 | 0.085 (0.015) | 1.5×10^-8^ |  | 0.060 (0.017) | 5.1×10^-4^ |  | 0.074 (0.011) | 5.6×10^-11^ |
| Eosinophil | rs2516399 | 6 | 31,589,278 | 6p21 | MHC region | A/G | 0.81 | - | 0.111 (0.019) | 4.4×10^-9^ |  | 0.095 (0.024) | 7.0×10^-5^ |  | 0.105 (0.015) | 1.8×10^-12^ |
| Eosinophil | rs9373124 | 6 | 135,464,902 | 6q23 | *HBS1L-MYB* | T/C | 0.65 | 0.96 | 0.101 (0.016) | 4.1×10^-10^ |  | 0.048 (0.020) | 0.015 |  | 0.080 (0.012) | 1.3×10^-10^ |
| Eosinophil | rs12670783 | 7 | 92,146,734 | 7q21 | *CDK6* | A/G | 0.53 | 0.98 | 0.071 (0.015) | 3.3×10^-6^ |  | 0.002 (0.018) | 0.90 |  | 0.043 (0.012) | 1.8×10^-4^ |
| Neutrophil | rs445 | 7 | 92,246,306 | 7q21 | *CDK6* | C/T | 0.69 | - | 0.074 (0.016) | 4.3×10^-6^ |  | 0.078 (0.019) | 4.5×10^-5^ |  | 0.076 (0.012) | 6.6×10^-10^ |
| Monocyte | rs1861113 | 7 | 93,721,303 | 7q21 | *COL1A2* | G/A | 0.77 | 0.77 | 0.089 (0.019) | 3.2×10^-6^ |  | 0.024 (0.022) | 0.27 |  | 0.061 (0.014) | 2.1×10^-5^ |
| Eosinophil | rs2642487 | 7 | 146,018,215 | 7q35 | *CNTNAP2* | C/A | 0.31 | - | 0.077 (0.016) | 2.6×10^-6^ |  | -0.023 (0.020) | 0.25 |  | 0.037 (0.012) | 0.0026 |
| Basophil | rs11761588 | 7 | 150,724,981 | 7q36 | *WDR86* | C/T | 0.87 | - | 0.107 (0.022) | 1.5×10^-6^ |  | -0.006 (0.027) | 0.82 |  | 0.061 (0.017) | 3.1×10^-4^ |
| Monocyte | rs10956483 | 8 | 130,641,292 | 8q24 | *MLZE* | C/G | 0.44 | 0.97 | 0.070 (0.015) | 2.0×10^-6^ |  | 0.072 (0.017) | 1.6×10^-5^ |  | 0.071 (0.011) | 2.1×10^-10^ |
| Basophil | rs2316799 | 9 | 90,710,882 | 9q22 | *EDG3* | A/C | 0.30 | 0.82 | 0.082 (0.018) | 4.7×10^-6^ |  | 0.007 (0.022) | 0.75 |  | 0.051 (0.014) | 2.1×10^-4^ |
| Basophil | rs11018874 | 11 | 89,515,085 | 11q14 | *NAALAD2* | G/A | 0.69 | 0.96 | 0.089 (0.017) | 8.2×10^-8^ |  | 0.062 (0.020) | 0.0017 |  | 0.077 (0.013) | 1.8×10^-9^ |
| Neutrophil | rs11056970 | 12 | 16,558,431 | 12p12 | *LMD3* | C/A | 0.83 | 0.96 | 0.090 (0.019) | 3.9×10^-6^ |  | -0.004 (0.023) | 0.88 |  | 0.052 (0.015) | 3.9×10^-4^ |
| Neutrophil | rs2188380 | 12 | 109,870,510 | 12q24 | *MYL2-CUTL2* | C/T | 0.21 | 0.71 | 0.099 (0.021) | 2.5×10^-6^ |  | 0.007 (0.025) | 0.77 |  | 0.062 (0.016) | 1.4×10^-4^ |
| Basophil | rs9550568 | 13 | 29,954,281 | 13q12 | *HMGB1* | T/C | 0.86 | - | 0.105 (0.022) | 9.8×10^-7^ |  | 0.023 (0.025) | 0.37 |  | 0.070 (0.017) | 2.6×10^-5^ |
| Monocyte | rs10147992 | 14 | 24,573,639 | 14q12 | *STXBP6* | G/A | 0.47 | 0.98 | 0.084 (0.014) | 5.5×10^-9^ |  | 0.029 (0.017) | 0.085 |  | 0.061 (0.011) | 1.2×10^-8^ |
| Neutrophil | rs4794822 | 17 | 35,410,238 | 17q21 | *PSMD3-CSF3* | T/C | 0.52 | 1.00 | 0.099 (0.015) | 4.5×10^-11^ |  | 0.085 (0.018) | 1.6×10^-6^ |  | 0.093 (0.011) | 4.0×10^-16^ |
| Eosinophil | rs8079078 | 17 | 63,251,628 | 17q24 | *BPTF* | C/A | 0.82 | - | 0.093 (0.020) | 3.0×10^-6^ |  | 0.031 (0.024) | 0.19 |  | 0.067 (0.015) | 1.1×10^-5^ |
| Lymphocyte | rs7237848 | 18 | 4,763,110 | 18p11 | no gene | T/C | 0.33 | 0.88 | 0.082 (0.017) | 7.6×10^-7^ |  | 0.048 (0.019) | 0.013 |  | 0.067 (0.013) | 1.5×10^-7^ |
| Basophil | rs8083155 | 18 | 62,501,525 | 18q22 | *CDH19* | T/A | 0.54 | 1.00 | 0.081 (0.015) | 6.2×10^-8^ |  | 0.005 (0.018) | 0.81 |  | 0.051 (0.012) | 1.4×10^-5^ |
| Lymphocyte | rs10404146 | 19 | 58,696,352 | 19q13 | *ZNF813-ZNF331* | A/T | 0.83 | 0.90 | 0.099 (0.021) | 3.8×10^-6^ |  | -0.040 (0.023) | 0.087 |  | 0.037 (0.016) | 1.8×10^-2^ |
| Eosinophil | rs7259060 | 19 | 60,943,102 | 19q13 | *NLRP9* | G/A | 0.44 | 0.98 | 0.093 (0.015) | 1.2×10^-9^ |  | -0.019 (0.018) | 0.30 |  | 0.048 (0.012) | 3.5×10^-5^ |
| Eosinophil | rs331615 | 20 | 55,071,944 | 20q13 | *BMP7* | T/A | 0.37 | 0.94 | 0.080 (0.016) | 6.8×10^-7^ |  | 0.032 (0.019) | 0.099 |  | 0.060 (0.012) | 9.4×10^-7^ |
| Basophil | rs7275212 | 21 | 38,774,421 | 21q22 | *ERG* | T/A | 0.08 | 0.93 | 0.191 (0.029) | 3.5×10^-11^ |  | 0.205 (0.035) | 6.4×10^-9^ |  | 0.197 (0.022) | 1.6×10^-18^ |

^a^SNPs that satisfied *P* < 5.0 × 10^-6^ in any of the GWASs for the WBC subtypes are indicated.

^b^The allele that increased the count of the corresponding WBC subtype in the GWAS was denoted as effect allele (EA) and is indicated based on the forward strand.

^c^Frequency of effect allele in the subjects enrolled in the GWAS.

^d^Imputation score of *Rsq* by MACH 1.0. For the genotyped SNP, "-" is indicated.

^e^Effect size of effect allele on the normalized trait.

WBC, white blood cell; GWAS, Genome-wide association study; SE, standard error.
