# Supplementary material for: Identification of Nine Novel Loci Associated with White Blood Cell Subtypes in a Japanese Population
Source: PLoS Genet. 2011 Jun 30;7(6):e1002067. doi: 10.1371/journal.pgen.1002067 (PMC3128095; doi:10.1371/journal.pgen.1002067)
Supplement: Table S4 — The associations in the previously-reported WBC subtype-associated loci. (DOC) [file pgen.1002067.s005.doc]

**Table S4.** The associations in the previously-reported WBC subtype-associated loci.

| WBC subtype | rsID | Chr | Position | Cytoband | Gene | EA/non-EA^a^ | No. subjects | Freq.^b^ | *Rsq*^c^ | Beta (SE)^c^ | *P* | ref. |
| --- | --- | --- | --- | --- | --- | --- | --- | --- | --- | --- | --- | --- |
| Eosinophil | rs1420101 | 2 | 102,324,148 | 2q11 | *IL1RL1* | T/C | 8,660 | 0.42 | - | 0.062 (0.015) | 4.8×10^-5^ | [2] |
| Eosinophil | rs12619285 | 2 | 213,532,290 | 2q34 | *IKZF2* | A/G | 8,660 | 0.32 | - | 0.048 (0.016) | 0.0027 | [2] |
| Eosinophil | rs4857855 | 3 | 129,743,240 | 3q21 | *GATA2* | C/T | 8,660 | 0.73 | - | 0.038 (0.017) | 0.026 | [2] |
| Eosinophil | rs2416257 | 5 | 110,463,389 | 5q22 | *WDR36* | C/T | 8,660 | 0.98 | - | 0.127 (0.052) | 0.015 | [2] |
| Eosinophil | rs4143832 | 5 | 131,890,876 | 5q23 | *IL5* | T/G | 8,660 | 0.18 | - | 0.028 (0.020) | 0.16 | [2] |
| Lymphocyte (CD4:CD8 ratio, CD8) | rs2524054 | 6 | 31,360,375 | 6p21 | MHC region | A/C | 8,668 | 0.16 | 0.95 | 0.056 (0.019) | 0.0037 | [3] |
| Eosinophil | rs2269426 | 6 | 32,184,477 | 6p21 | MHC region | G/A | 8,660 | 0.67 | - | 0.005 (0.016) | 0.77 | [2] |
| Lymphocyte (CD4) | rs9271366 | 6 | 32,694,832 | 6p21 | MHC region | G/A | 8,668 | 0.18 | - | 0.023 (0.019) | 0.23 | [3] |
| Eosinophil | rs9494145 | 6 | 135,474,245 | 6q23 | *HBS1L-MYB* | T/C | 8,660 | 0.68 | - | 0.073 (0.016) | 6.5×10^-6^ | [2] |
| Eosinophil | rs748065 | 8 | 21,734,049 | 8p21 | *GFRA2* | A/G | 8,660 | 0.42 | - | 0.003 (0.015) | 0.83 | [2] |
| Eosinophil | rs3939286 | 9 | 6,200,099 | 9p24 | *IL33* | C/T | 8,660 | 0.97 | - | 0.006 (0.045) | 0.89 | [2] |
| Monocyte | rs7023923 | 9 | 112,965,355 | 9q31 | *LPAR1* | T/C | 8,653 | 0.81 | 0.94 | 0.035 (0.019) | 0.067 | [4] |
| Eosinophil | rs3184504 | 12 | 110,368,991 | 12q24 | *SH2B3* | T/C | 8,660 | 0.00 | N.A. | N.A. | N.A. | [2] |
| Lymphocyte (NK cells) | rs1838149 | 17 | 30,843,415 | 17q12 | *SLFN13-PEX12* | G/A | 8,668 | 0.93 | 0.79 | 0.060 (0.033) | 0.069 | [3] |
| Neutrophil | rs4794822 | 17 | 35,410,238 | 17q21 | *PSMD3-CSF3* | T/C | 8,788 | 0.52 | 1.00 | 0.099 (0.015) | 4.5×10^-11^ | [5] |
| Neutrophil | rs2072910 | 20 | 9,313,303 | 20p12 | *PLCB4* | T/C | 8,788 | 0.70 | 1.00 | 0.067 (0.016) | 3.9×10^-5^ | [5] |

^a^The allele that increased the count of the corresponding WBC subtype in the study population was denoted as effect allele and indicated based on forward strand.

^b^Frequency of effect allele in the subjects in the study.

^c^Imputation score of *Rsq* by MACH 1.0. For the genotyped SNP, "-" is indicated.

^d^Effect size of effect allele on the normalized trait.

WBC, white blood cell; SE, standard error; N.A., not available.
