# Supplementary material for: Identification of Nine Novel Loci Associated with White Blood Cell Subtypes in a Japanese Population
Source: PLoS Genet. 2011 Jun 30;7(6):e1002067. doi: 10.1371/journal.pgen.1002067 (PMC3128095; doi:10.1371/journal.pgen.1002067)
Supplement: Table S5 — Characteristics and distributions of traits in the study populations by the CHARGE Consortium. (DOC) [file pgen.1002067.s006.doc]

**Table S5.** Characteristics and distributions of traits in the study populations by the CHARGE Consortium.

|  | Cohorts enrolled in CHARGE Consortium | | | | | | |  |  |
| --- | --- | --- | --- | --- | --- | --- | --- | --- | --- |
|  | **AGES** | **ARIC** | **BLSA** | **FHS** | **Health ABC** | **InChianti** | **RS** | No. subjects^a^ | Transformation |
| Total No. subjects | 3,217 | 4,846 | 337 | 3,909 | 1,075 | 1,014 | 5,111 | - | - |
| Age (mean ± SD) | 76.4 (5.5) | 54.3 (5.7) | 66.8 (13.9) | 35.9 (10.4) | 75.7 (2.8) | 68.1 (15.3) | 69.1 (9.0) | - | - |
| Female (%) | 58.0 | 53.2 | 48.7 | 51.2 | 47.1 | 57.0 | 59.5 | - | - |
| Smoking (%) | 12.7 | 21.1 | 2.7 | 42.2 | 4.3 | 17.7 | 22.6 | - | - |
| Neutrophil (×10^3^/μL) | 3.51 (1.30) | 3.65 (1.12) | 3.15 (0.87) | N.A. | 3.66 (1.01) | 3.63 (1.02) | N.A. | 10,489 | natural log |
| Lymphocyte (×10^3^/μL) | 1.73 (0.94) | 1.81 (0.48) | 1.64 (0.45) | N.A. | 1.74 (0.58) | 1.83 (0.53) | 2.50 (0.78) | 15,600 | square-root |
| Monocyte (×10^3^/μL) | 0.54 (0.18) | 0.34 (0.14) | 0.41 (0.15) | N.A. | 0.53 (0.15) | 0.31 (0.09) | N.A. | 10,489 | natural log |
| Basophil (×10^3^/μL) | 0.029 (0.025) | 0.025 (0.033) | 0.012 (0.015) | N.A. | 0.060 (0.031) | 0.026 (0.019) | N.A. | 10,489 | square-root |
| Eosinophil (×10^3^/μL) | 0.207 (0.144) | 0.104 (0.103) | 0.174 (0.093) | N.A. | 0.173 (0.102) | 0.171 (0.091) | N.A. | 10,489 | square-root |

Characteristics of the subjects and the distribution of white blood cell subtypes in the study conducted by the CHARGE Consortium [30].

Details of the study is described elsewhere [23].

^a^For each of the traits, the subjects with values beyond ± 3 SD of the population mean were excluded.

N.A., data not available.
