# Supplementary material for: Identification of Nine Novel Loci Associated with White Blood Cell Subtypes in a Japanese Population
Source: PLoS Genet. 2011 Jun 30;7(6):e1002067. doi: 10.1371/journal.pgen.1002067 (PMC3128095; doi:10.1371/journal.pgen.1002067)
Supplement: Table S6 — Characteristics and distributions of the traits enrolled in the pleiotropic association study. (DOC) [file pgen.1002067.s007.doc]

**Table S6.** Characteristics and distributions of the traits enrolled in the pleiotropic association study.

| Traits | No. subjects | Mean ± SD | Unit | Transformation^a^ |
| --- | --- | --- | --- | --- |
| WBC | 28,795 | 6.01 ± 1.89 | ×10^3^/μL | Common log |
| RBC | 28,981 | 4.23 ± 0.54 | ×10^6^/μL | None |
| Hb | 30,156 | 13.0 ± 1.7 | g/dL | None |
| Ht | 30,230 | 39.1 ± 4.7 | % | None |
| MCH | 28,977 | 31.5 ± 2.0 | fL | None |
| MCHC | 28,956 | 33.2 ± 1.0 | pg | None |
| MCV | 28,756 | 93.0 ± 5.8 | % | None |
| PLT | 28,264 | 22.7 ± 7.5 | ×10^4^/μL | Common log |

^a^After the transformation, each trait was normalized. The subjects with values beyond ± 4 SD after the normalization were excluded.

Subjects consisted of Japanese patients in 27 disease groups as follows: drug eruption (n = 3,553), Diabetes Mellitus (n = 2,854), rheumatoid arthritis (n = 2,166), myocardial infarction (n = 1,625), breast cancer (n = 1,540), gastric cancer (n = 1,540), lung cancer (n = 1,537), colorectal cancer (n = 1,525), prostate cancer (n = 1,505), fibroid of uteris (n = 1,505), osteoporosis (n = 1,484), ischemic stroke (n = 1,432), cirrhosis (n = 1,365), warfarin intake (n = 1,316), endometriosis (n = 1,261), liver cancer (n = 1,161), peripheral artery disease (n = 855), atrial fibrillation (n = 811), gallbladder/bile duct cancers (n = 251), pancreatic cancer (n = 243), tuberculosis (n = 162), cervical cancer of uteris (n = 141), hepatitis B (n = 136), uterine body cancer (n = 136), esophageal cancer (n = 124), keloid (n = 119), and ovarian cancer (n = 107).

WBC, total white blood cell count; RBC, red blood cell count; Hb, hemoglobin; Ht, hematocrit; MCV, mean corpuscular volume; MCH, mean corpuscular hemoglobin; MCHC, mean corpuscular hemoglobin concentration; PLT, platelet count.
