# Supplementary material for: Identification of Nine Novel Loci Associated with White Blood Cell Subtypes in a Japanese Population
Source: PLoS Genet. 2011 Jun 30;7(6):e1002067. doi: 10.1371/journal.pgen.1002067 (PMC3128095; doi:10.1371/journal.pgen.1002067)
Supplement: Text S1 — Full descriptions of acknowledgements. (DOC) [file pgen.1002067.s008.doc]

**Supplementary Information**

**Acknowledgements**

We would like to thank all the staff of the Laboratory for Statistical Analysis at RIKEN for their technical assistance. This study was supported by Ministry of Education, Culture, Sports, Science and Technology, Japan.

The authors acknowledge the essential role of the Cohorts for Heart and Aging Research in Genome Epidemiology (CHARGE) Consortium in development and support of this manuscript. CHARGE members include the Rotterdam Study (RS), Framingham Heart Study (FHS), Cardiovascular Health Study (CHS), the NHLBI’s Atherosclerosis Risk in Communities (ARIC) Study, and the NIA’s Iceland Age, Gene/Environment Susceptibility (AGES) Study. The collaboration of studies such as the Health Aging and Body Composition Study (Health ABC), the Baltimore Longitudinal Study of Aging (BLSA), the Invecchiare in Chianti Study (InChianti), and the Heart and Vascular Health Study (HVH) also played a vital role.

This research was made possible by NIA/NIH contract AG000932‐02 (2009) Characterization of Normal Genomic Variability. This study utilized the high‐performance computational capabilities of the Biowulf Linux cluster at the National Institutes of Health, Bethesda, MD [http://biowulf.nih.gov].

The Age, Gene/Environment Susceptibility Reykjavik Study is funded by NIH contract N01‐AG‐12100, the NIA Intramural Research Program, Hjartavernd (the Icelandic Heart Association) and the Althingi (the Icelandic Parliament).

The Atherosclerosis Risk in Communities Study is carried out as a collaborative study supported by National Heart, Lung, and Blood Institute contracts N01‐HC‐55015, N01‐HC‐55016, N01‐HC‐ 55018, N01‐HC‐55019, N01‐HC‐55020, N01‐HC‐55021, N01‐HC‐55022, and grants R01HL087641, R01HL59367 and R01HL086694; National Human Genome Research Institute contract U01HG004402; and National Institutes of Health contract HHSN268200625226C. The authors thank the staff and participants of the ARIC study for their important contributions. Infrastructure was partly supported by Grant Number UL1RR025005, a component of the National Institutes of Health and NIH Roadmap for Medical Research.

The National Heart, Lung, and Blood Institute's Framingham Heart Study is a joint project of the National Institutes of Health and Boston University School of Medicine and was supported bythe National Heart, Lung, and Blood Institute's Framingham Heart Study (contract No. N01‐HC‐25195) and its contract with Affymetrix, Inc. for genotyping services (contract No. N02‐HL‐6‐4278). Analyses reflect the efforts and resource development from the Framingham Heart Study investigators participating in the SNP Health Association Resource (SHARe) project. A portion of this research was conducted using the Linux Cluster for Genetic Analysis (LinGA‐II) funded by the Robert Dawson Evans Endowment of the Department of Medicine at Boston University School of Medicine and Boston Medical Center.

The Health ABC Study was supported in part by the Intramural Research Program of the NIH, National Institute on Aging, NIA contracts N01AG62101, N01AG62103, and N01AG62106. The genome‐wide association study was funded by NIA grant 1R01AG032098‐01A1 to Wake Forest University Health Sciences and genotyping services were provided by the Center for Inherited Disease Research (CIDR). CIDR is fully funded through a federal contract from the National Institutes of Health to The Johns Hopkins University, contract number HHSN268200782096C.

The InChianti Study was supported as a "targeted project" (ICS 110.1RS97.71) by the Italian Ministry of Health, by the U.S. National Institute on Aging (Contracts N01‐AG‐916413, N01‐AG‐821336, 263 MD 9164 13, and 263 MD 821336) and in part by the Intramural Research Program, National Institute on Aging, National Institutes of Health, USA.

Rotterdam Study GWAS database of the Rotterdam Study was funded through the Netherlands Organization of Scientific Research NWO (nr. 175.010.2005.011, 911.03.012) and the Research Institute for Diseases in the Elderly (RIDE). This study was supported by the Netherlands Genomics Initiative (NGI)/NWO project number 050 060 810 (Netherlands Consortium for Healthy Ageing). We thank Dr Michael Moorhouse, Pascal Arp, Mila Jhamai, Marijn Verkerk and Sander Bervoets for their help in creating the genetic database. We thank the laboratory technicians Jeannette M Vergeer ‐ Drop, Bernadette H M van Ast ‐ Copier, Andy A L J van Oosterhout, Sue Ellen Mauricia, Andrea J M Vermeij ‐ Verdoold, Els Halbmeijer ‐ van der Plas, Debby M S Lont, and Hasna Kariouh for their help in phenotype assessment. The Rotterdam Study is supported by the Erasmus Medical Center and Erasmus University, Rotterdam; the Netherlands organization for scientific research (NWO), the Netherlands Organization for the Health Research and Development (ZonMw), the Research Institute for Diseases in the Elderly (RIDE), the Netherlands Heart Foundation, the Ministry of Education, Culture and Science, the Ministry of Health, Welfare and Sports, the European Commission (DG XII), and the Municipality of Rotterdam.

The funders had no role in study design, data collection and analysis, decision to publish, or preparation of the manuscript.
